# MicroRNA-106b inhibits osteoclastogenesis and osteolysis by targeting RANKL in giant cell tumor of bone

**DOI:** 10.18632/oncotarget.4223

**Published:** 2015-05-22

**Authors:** Ting Wang, Huabin Yin, Jing Wang, Zhenxi Li, Haifeng Wei, Zhi'an Liu, Zhipeng Wu, Wangjun Yan, Tielong Liu, Dianwen Song, Xinghai Yang, Quan Huang, Wang Zhou, Jianru Xiao

**Affiliations:** ^1^ Department of Bone Tumor Surgery, Changzheng Hospital, Second Military Medical University, Shanghai, China; ^2^ Department of Anatomy, Xuzhou Medical College, Xuzhou, China

**Keywords:** giant cell tumor of bone, miR-106b, RANKL, osteoclastogenesis, osteolysis

## Abstract

Giant cell tumor (GCT) of bone consists of three major cell types: giant cells, monocytic cells, and stromal cells. From microarray analysis, we found that miR-106b was down-regulated in GCT clinical samples and further determined by fluorescence in situ hybridization. In addition, the expression of novel potential target of miR-106b, RANKL, was elevated in GCT along with previously determined targets in other tumors such as IL-8, MMP2 and TWIST. In a RANKL 3′UTR luciferase reporter assays, agomiR-106b repressed the luciferase activity and the effect was eliminated when the targeting site in the reporter was mutated, suggesting a direct regulation of miR-106b on RANKL mRNA. Moreover, overexpression of miR-106b in GCTSCs through TALEN-mediated site-specific knockin clearly inhibited osteoclastogenesis and osteolysis. By grafting the GCT onto the chick CAM, we confirmed the inhibitory effect of miR-106b on RANKL expression and giant cell formation. Furthermore, in an OVX mouse model, silencing of miR-106b increased RANKL protein expression and promoted bone resorption, while up-regulation of miR-106b inhibited bone resorption. These results suggest that miR-106b is a novel suppressor of osteolysis by targeting RANKL and some other cytokines, which indicates that miR-106b may be a potential therapeutic target for the treatment of GCT.

## INTRODUCTION

Giant cell tumor (GCT) of bone, which accounts for approximately 6% of all primary bone tumors [1], is characterized by extensive bone resorption [2], leading to regional pain and the predisposition to pathologic fractures [3]. Although considered as a non-cancerous tumor [4], GCT is known for potential recurrence following treatment with a reported incidence rate between 18% and 50% [5]. Histologically, GCT consists of three major cell types: multinucleated osteoclast-like giant cells, monocytic round-shaped macrophage-like cells, and spindle-shaped fibroblast-like stromal cells, also known as giant-cell tumor stromal cells (GCTSCs) [6]. Giant cell formation is thought to occur in a similar manner as osteoclastogenesis, where osteoblasts express the receptor activator of nuclear factor-kB ligand (RANKL), which stimulates its receptor, RANK, on osteoclast precursor cells and initiates their fusion into osteoclasts [7, 8]. Proliferating GCTSCs are believed to be the neoplastic component of GCT which is known to express RANKL [9] and thought to stimulate giant cell formation from RANK-expressing monocytic cells [10].

RANKL, a member of the tumor necrosis factor (TNF) family, triggers osteoclastogenesis by forming a complex with RANK, a member of the TNF receptor family [11]. RANKL binds to RANK expressed on the surface of osteoclast precursor cells and drives them to form active multinucleated osteoclasts for bone resorption during bone remodeling [4], while osteoprotegerin (OPG) competes with RANK, resulting in the inhibition of osteoclastic bone destruction [8]. A functional interaction between RANKL and RANK is necessary for osteoclast differentiation, survival, and activation. In addition, the up-regulation of RANKL-RANK signaling also plays an important role in other osteolysis-related diseases such as osteoporosis [12] and osteofibrousdysplasia [13], as well as bone metastasis [14]. Recent findings have revealed a number of transcription factors that regulate RANKL expression, such as parathyroid hormone (PTH) [15] and vitamin D3 [16]. However, as another vital process for the final protease activity, post-transcriptional regulation of RANKL remains poorly understood.

MicroRNAs (miRNAs) are a class of abundant, approximately 22 nucleotide non-coding RNAs that mediate post-transcriptional regulation of target mRNAs [17]. By binding to the 3′-untranslated regions (3′UTRs) of specific mRNAs, miRNAs repress translation or degrade target mRNAs [18]. Bioinformatics have predicted that miRNAs have the capacity to regulate about one-third of all mammalian genes [19], and numerous studies have linked aberrant miRNA expression to pathological conditions such as cancer [17]. Despite this, the function of miRNA-induced osteolysis in GCT has rarely been reported. Given the similar forming process of giant cells in GCT and osteoclasts, miRNAs that regulate osteoclastogenesis may play a key role in GCT. Emerging evidence has revealed a general necessity for miRNAs in osteoclastogenesis, knowing that ectopic expression of miR-148a and miR-503 inhibited the expression of V-maf musculoaponeurotic fibrosarcoma oncogene homolog B (MAFB) [20] and RANK [21], resulting in the blockage of osteoclast differentiation. Our previous studies have confirmed that miR-126-5p played a role in osteoclastogenesis through targeting MMP13 and PTHrP in GCT [22, 23]. Although miRNAs in osteoclast differentiation are widely recognized, the function of miRNAs related to RANKL, which is pivotal for osteoclastogenesis, still remains elusive.

To explore the role of miRNAs during the process of osteolysis in GCT, miRNA expression changes between GCT and normal cancellous bone were compared, and miR-106b was found to be down-regulated in GCT. Furthermore, the cellular and molecular functions of miR-106b and one of its key targets, RANKL, were elucidated. Lastly, we validated these observations *in vivo* using a short short-term GCT model of chick chorio-allantoic membrane (CAM) and the OVX mice model, and provide data in support of targeting the miR-106b∼RANKL axis in preventing giant cell formation and osteolysis.

## RESULTS

### MiR-106b is down-regulated significantly in GCT

**Figure 1:**
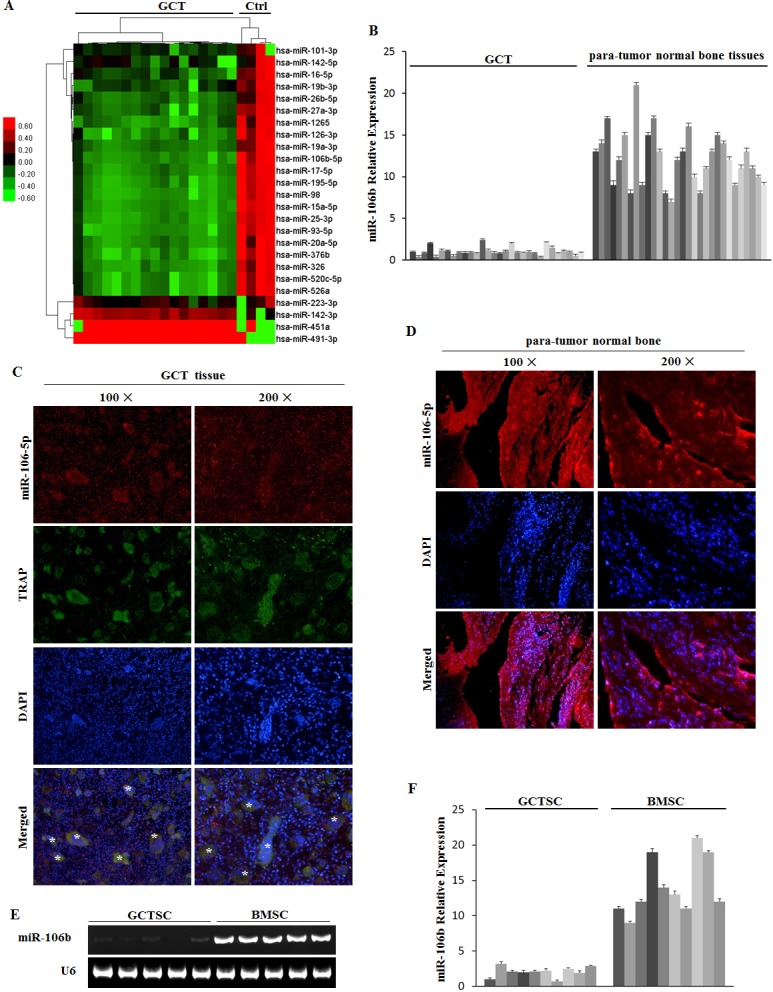
microRNA regulation in GCT tissue **A.** Microarray assays in GCT and normal bone tissues. **B.** qRT-PCR measurement of miR-106b levels in tumor and normal bone tissues from the 30 GCT patients. **C.** MiRNA-106b (red) and TRAP (green) detected by FISH and IF in GCT specimens. **D.** MiRNA-106b (red) detected by FISH in para-tumor normal bone tissue specimens. **E.** PCR assay of miR-106b was performed in GCTSCs and BMSCs. **F.** The levels of miR-106b in GCTSCs and BMSCs were detected by qRT-PCR assay.

Human microarray assays of GCT samples (*n* = 17) and non-tumor infected cancellous bones (*n* = 4) were performed (Figure 1A, Supplemental Table 1). Bioinformatics analysis was applied to the data set of these differentially regulated miRNAs (Supplemental Figure 1). According to the results, we focused on miR-106b for its key role in tumor progression [24, 25] and its potential relevance to osteolysis. To validate the microarray data, we further detected the expression of miR-106b in 30 clinical GCT tissues and 30 normal cancellous bone tissues (Table 1, Supplemental Table 1) and the results were consistent with those of miRNA microarray (Figure 1B). The results of fluorescence in situ hybridization (FISH) further confirmed that the expression of miR-106b was down-regulated in GCT of bone (Figure 1C, 1D). Moreover, we isolated GCTSCs and bone marrow mesenchymal stem cells (BMSCs) from part of the GCT patients and detected the level of miR-106b. The result showed that the level of miR-106b in GCTSCs was significantly lower than it in BMSCs (Figure 1E, 1F).

**Table 1:** Characteristics of the 30 GCT patients

	GCT of bone patients
Age (years)	33.2±10.9
Sex (male/femal)	12/18
Disease history (months)	25.4±34.9
Tumor size (cm)	7.4±2.8
Tumor site (spine/limbs)	29/1
Primary/recurrence	17/13
Rseection (total/subtotal)	24/6

### The target genes of miR-106b are overexpressed in GCT tissues

**Figure 2:**
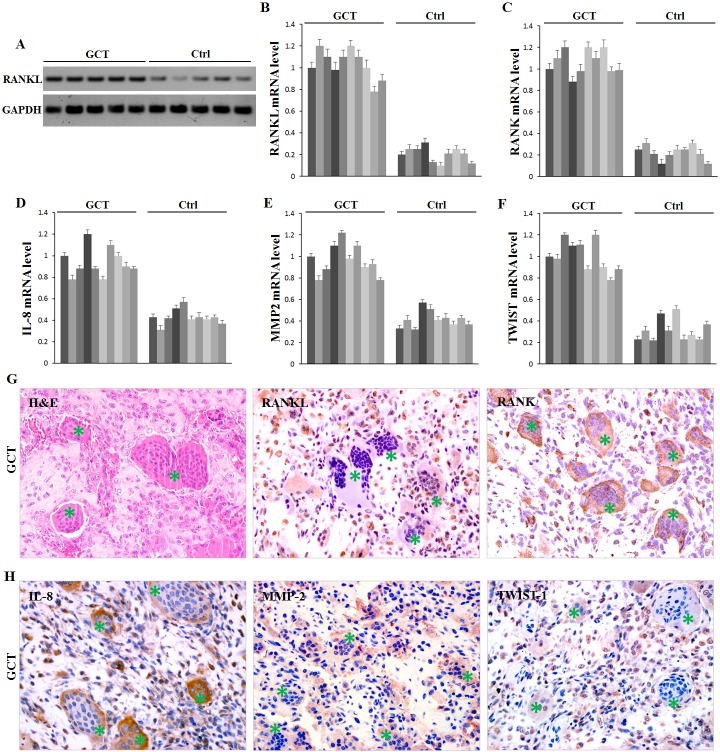
Expression of possible target proteins of miR-106b in GCT tissue **A.** Western Blot measurement of RANKL protein. **B.**-**F.** qRT-PCR measurement of mRNA levels of RANKL, RANK, IL-8, MMP2 and TWIST1. **G.**, **H.** H&E staining and IHC staining of RANKL, RANK, IL-8, MMP2 and TWIST1 in human GCT tissue specimens. Giant cells are marked with asterisks.

The above bioinformatics analysis also identified that some cytokines involved in osteolysis were possible target genes of miR-106b, including RANKL, IL-8, MMP2 and TWIST. Additionally, literature search convinced the regulation of IL-8, MMP2 and TWIST by miR-106b in other tumors [26-28], and their overexpression in GCT has already been confirmed [29, 30]. Western blot assay in our study confirmed that RANKL was up-regulated in GCT (Figure 2A). Using qRT-PCR assay, mRNA levels of RANKL, RANK, IL-8, MMP2 and TWIST in GCT tissues were confirmed to be evaluated in GCT compared with those in cancellous bones (Figure 2B-2F). To further investigate the distribution of these cytokines, immunohistochemistry was performed on paraffin-embedded clinical GCT samples. The results showed that RANKL, MMP2 and TWIST were predominantly localized in GCTSCs, while RANK and IL-8 were expressed both in GCTSCs and in giant cells (Figure 2G, 2H). Meanwhile, the expression of OPG, an important inhibitor of RANKL function, had no clear difference between GCT and control tissues (Supplemental Figure 2A).

### MiR-106b directly targets RANKL

**Figure 3:**
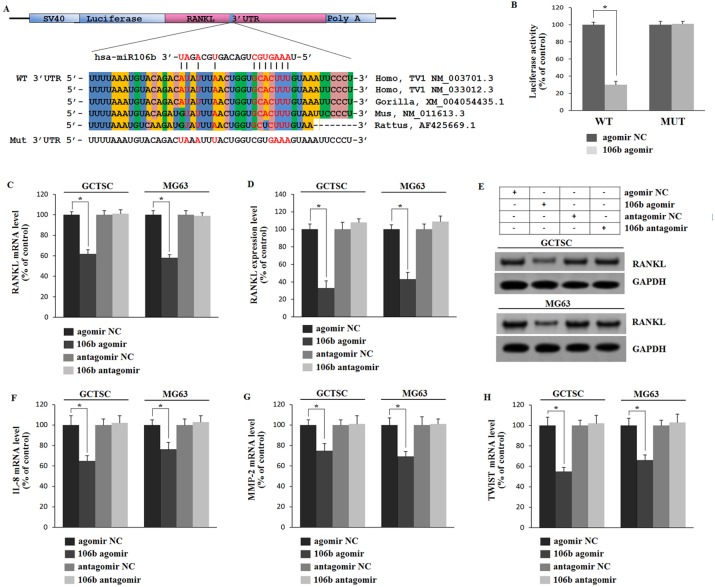
Mir-106b directly targets RANKL and regulates IL-8, MMP2 and TWIST **A.** Prediction of the miR-106b target site in RANKL 3′UTR in humans and animals. Schematic diagram of miR-106b with WT and MUT 3′UTR regions of RANKL are shown in complementary pairing. Mutated nucleotides are labeled. **B.** Determination of the effect of agomir-Control, agomiR-106b on luciferase activity in HEK293 cells transfected with either WT-RANKL 3′UTR reporter or mutant RANKL 3′ UTR reporter. Firefly luciferase values are normalized for Renilla luciferase. **C.** qRT-PCR analysis of RANKL mRNA levels in GCTSCs and MG63 cells treated with agomiR-106b or antagomiR-106b and their corresponding negative controls. **D.**, **E.** ELISA and Western blot analysis of RANKL protein level. **F.**-**H.** qRT-PCR analysis of the mRNA levels of MMP2, IL-8 and TWIST. *P < 0.05.

It has been demonstrated that miRNA acts primarily through binding to the 3′untranslated regions (3′UTRs) of mRNA resulting in degradation and translation repression [17]. We explicitly predicted that the binding site of miR-106b was in 3′UTR of RANKL mRNA and examined it by luciferase assay. A luciferase reporter construct containing the 3′UTR of RANKL was generated and mutations were introduced into the predicted binding site (Figure 3A). The luciferase expression vectors containing the 3′UTR of RANKL (WT) or the mutation sequences (MUT) with agomiR-106b were introduced into HEK293 cells and the effect of miR-106b was measured on luciferase translation by the level of luciferase enzyme activity. The results showed that the luciferase activity of the RANKL 3′UTR reporter gene was significantly suppressed in WT group after being stimulated by agomiR-106b, while mutation abolished this repression, confirming that miR-106b could suppress RANKL expression by binding to this site of the 3′UTR (Figure 3B).

To identify the action of miR-106b on RANKL, IL-8, MMP2 and TWIST we transfected GCTSCs and MG63 cells, a cell line of osteosarcoma known to express these cytokines [31, 32], with agomiR-106b or antagomiR-106b and measured the mRNA and protein levels of RANKL by qRT-PCR, Western blot and ELISA. Relative to the controls, RANKL protein levels were reduced significantly after agomir transfection in both cell types, while RANKL mRNA levels showed a similar tendency (Figure 3C-3E). However, the mRNA levels exhibited less fluctuant as compared with the protein levels. By using the qRT-PCR assay, we found that the mRNA levels of IL-8, MMP2 and TWIST exhibited analogous changes after agomiR-106b and antagomiR-106b transfection (Figure 3F-3H), while the mRNA level of OPG showed no clear fluctuation after the transfection (Supplemental Figure 2B). These results suggest that miR-106b could down-regulate RANKL expression by interacting with 3′UTRs binding site of RANKL, and also inhibit MMP2, IL-8 and TWIST expression.

### MiR-106b regulates osteoclastogenesis through targeting RANKL, IL-8, MMP2 and TWIST

**Figure 4:**
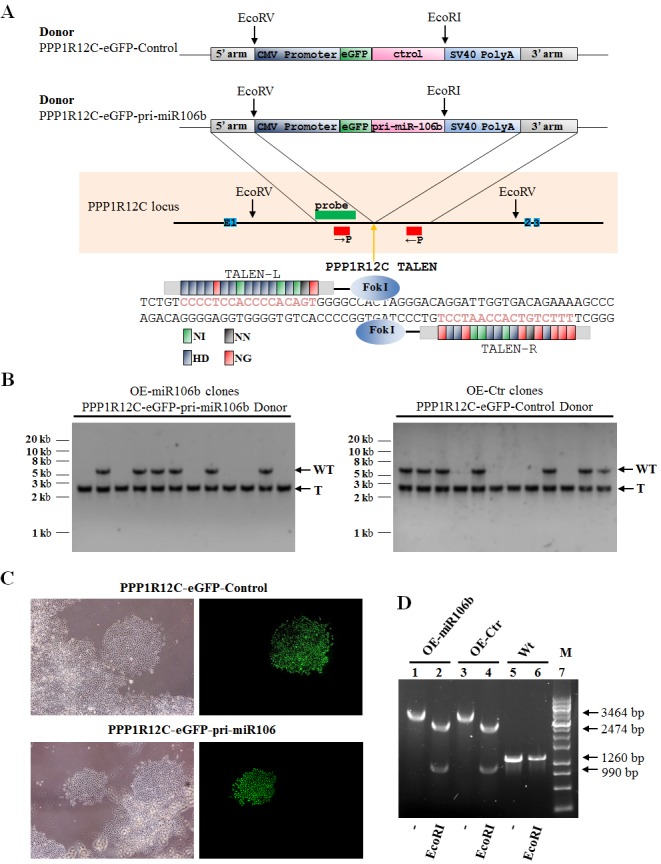
Genetic engineering of GCTSCs using TALENs **A.** Schematic overview depicting the targeting strategy for PPP1R12C. Primers are shown as red boxes; southern blot probes as green box; exons as blue boxes. The orange arrow indicates the cut site by the TALENs. Donor plasmids: CMV Promoter,human cytomegalovirus (CMV) immediate early promoter gene; eGFP, enhanced green fluorescent protein gene; Pri-miR-106b, Pri-miR106b gene; Control, Pri-miR106 binding site mutant gene; SV40 Poly A, SV40 early mRNA polyadenylation signal gene. Below, scheme of PPP1R12C TALENs and their recognition sequence. TALE repeat domains are colored to indicate the identity of the repeat variable diresidue (RVD); each RVD is related to the cognate targeted DNA base by the following code (NI = A, HD = C, NN = G, NG = T). **B.** Southern blot analysis of OE-miR106b (Overexpression miR-106b) and OE-control (Overexpression miR-106b mutant) GCTSCs targeted using PPP1R12C TALENs and the PPP1R12C-eGFP-pri-miR106b or PPP1R12C-eGFP-Control donor plasmids. Genomic DNA was digested with EcoRV and hybridized with an^32^P-labeled probe (left). The probe detects a 5.4 kb WT and a 2.6 kb targeted fragment. WT, wild type; T, correctly targeted allele. **C.** Phase contrast images and eGFP fluorescence ofPPP1R12C-eGFP-pri-miR106b or PPP1R12C-eGFP-Control targeted GCTSCs clones. **D.** Genomic PCR and restriction digestion characterization of OE-miR106b and OE-control GCTSCs.

**Figure 5:**
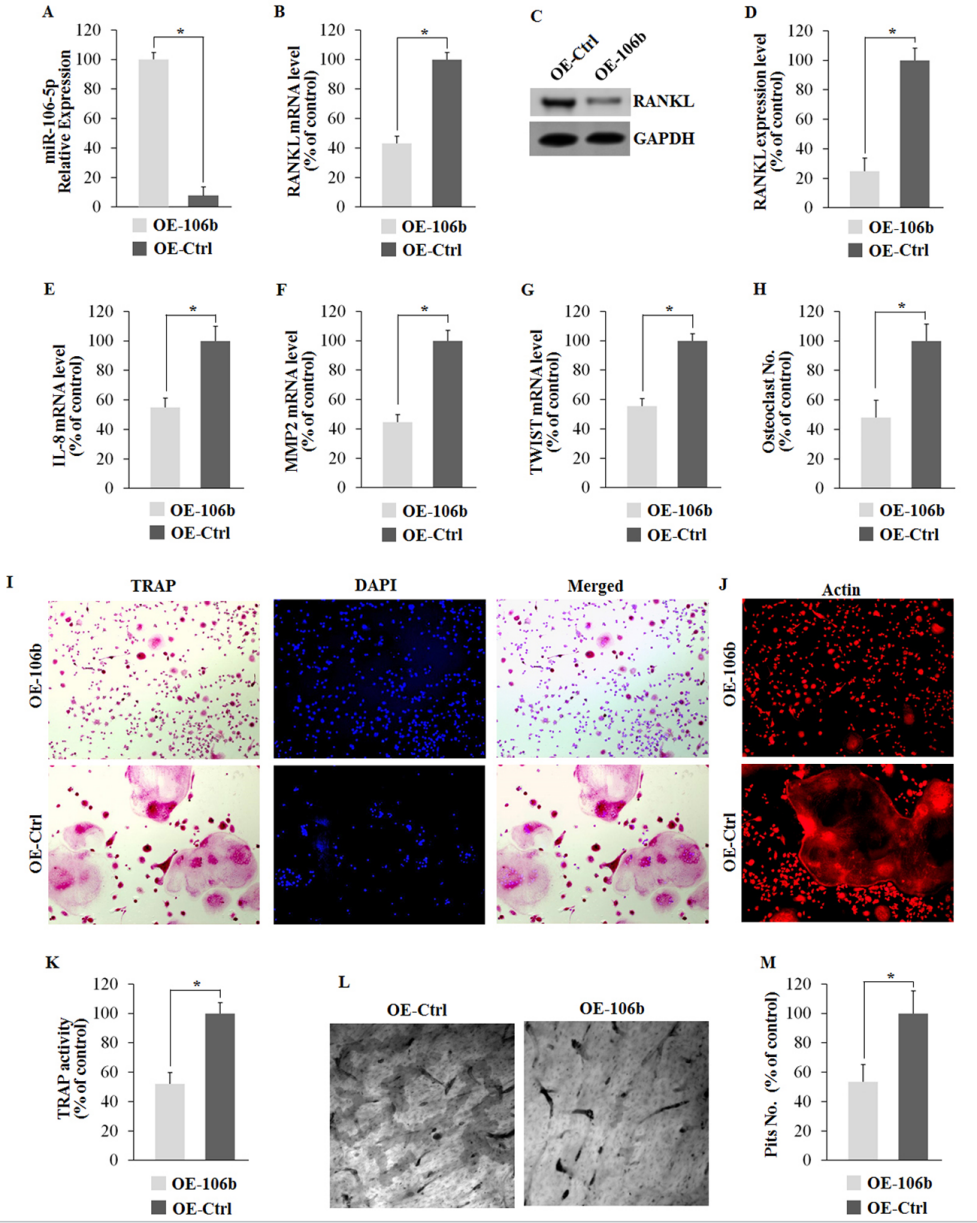
Overexpression of miR-106b suppresses its target genes and osteoclastogenesis *in vitro* **A.** qRT-PCR analysis of miR-106b level 48 h after transfection. **B.** qRT-PCR analysis of mRNA level of RANKL. **C.**, **D.** Western blot and ELISA analysis of RANKL protein level. **E.**-**G.** qRT-PCR analysis of mRNA levels of IL-8, MMP2 and TWIST. (H-M) Mouse BMMs were seeded on dentin slices or empty wells and cultured with conditional medium containing MCFS (10 ng/mL) from OE-miR106b or OE-miR106b-Control GCTSCs for 7 days. **H.** The number of TRAP positive osteoclasts per well. **I.**TRAP staining of BMM cells. **J.** Phalloidin-Rhodamine staining of BMM cells. **K.** TRAP activity assay of BMM cells. **L.** Dentine slices stained with Mayer's hematoxylin after removal of cells. **M.** The number of pits per dentine slice. *P < 0.05.

To gain a more comprehensive understanding of the regulatory role of miR-106b in RANKL-RANK signaling and osteolysis, we established the stable cell lines (OE-miR-106b and OE-control) using TALENs targeting the PPP1R12C (the AAVS1 locus), which is considered to have no relevance to a known pathophysiology [33], and corresponding donor plasmids bearing homologous sequences. Briefly, GCTSCs were transfected with two TANEN vectors in conjunction with a targeting vector containing the EGFP gene and DNA fragments of pri-miR-106b (Figure 4A). Clones with a successful recombination in EGFP-Pri-miR-106b and EGFP-Pri-miR-106b-mutant were identified by genomic PCR and restriction digestion (Figure 4B-4D). By using qRT-PCR assay, we confirmed that OE-miR-106b cells expressed much higher level of miR-106b than OE-control cells (Figure 5A). Meanwhile, the mRNA and protein levels of RANKL were both down-regulated markedly in OE-miR-106b GCTSCs (Figure 5B-5D). Furthermore, the mRNA levels of IL-8, MMP2 and TWIST also exhibited opposite changes as compared with miR-106b expression (Figure 5E-5G), while no obvious change of OPG mRNA level was observed (Supplemental Figure 2C).

To investigate the role of miR-106b in osteolysis, we selected BMMs with M-CSF stimulation as in the vitro osteoclast (OC) differentiation model. OE-miR-106b and OE-control GCTSCs were used as different stimuli during bone resorption and osteoclast precursor differentiation. The two groups of BMMs were cultured with conditional medium containing M-CFS (10 ng/mL) from OE-miR-106b or OE-Control GCTSCs respectively. Seven days after, BMMs treated with the conditional medium of OE-miR-106b GCTSCs exhibited much less formation of tartrate resistant acid phosphatase (TRAP)-positive multinucleated osteoclasts as compared with the control (Figure 5H, 5I). TRAP activity was also inhibited in OE-miR-106b group (Figure 5K). Actin ring formation assays, another obvious characteristic of mature osteoclasts during osteoclastogenesis and a prerequisite for osteoclast bone resorption [34], similarly supported that the overexpression of miR-106b in GCTSCs suppressed the osteoclastogenesis (Figure 5J). In addition, the number and area of pits on the surface of the dentin slices were markedly decreased in OE-miR-106b conditional mediums group (Figure 5L, 5M). These results suggest that miR-106b played a negative role in osteoclastogenesis, and RANKL, IL-8, MMP2 and TWIST were the possible targets of miR-106b during this process.

### Mir-106b regulates the expression of RANKL and giant cell formation in the GCT model of chick CAM

**Figure 6:**
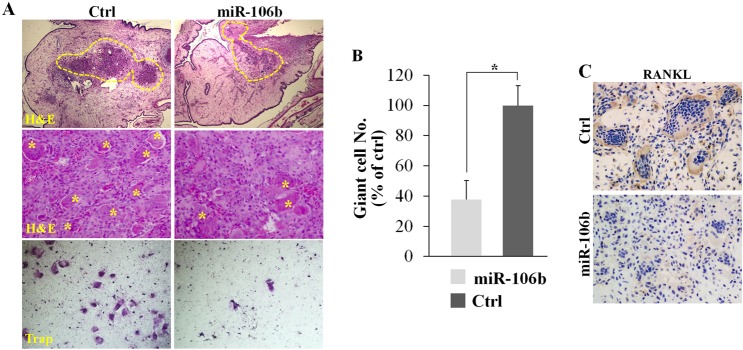
MiR-106b inhibits giant cell formation and the expression of RANKL in GCT *in vivo* Re-suspended tumor suspensions were deposited into the CAM of eggs and the tumors were picked out for further experiments at day 16. **A.** H&E staining and TRAP staining of the tumors. **B.** The number of TRAP+ giant cells per field. **C.** IHC staining of RANKL of the tumors. *P < 0.05.

Due to the limited proliferation ability and complex composition of GCT, it is difficult to an *in vivo* experiment of GCT. In fact, the only animal model reported before was a short-term GCT model of chick CAM [35]. We used this animal model to detect the role of miR-106b in GCT *in vivo*. Tumor cells were injected into the CAM, while agomiR-106b or PBS was injected every two days. After six days, tumors were picked out for H&E staining, TRAP staining or RANKL immunohistochemical staining. The result showed that eggs with agomiR-106b had significantly fewer TRAP+ giant cells as compared with the controls (Figure 6A, 6B). Meanwhile, RANKL expression was clearly lower in tumors injected with agomiR-106b as compared with the controls (Figure 6C).

### MiR-106b regulates bone resorption in OVX mice

**Figure 7:**
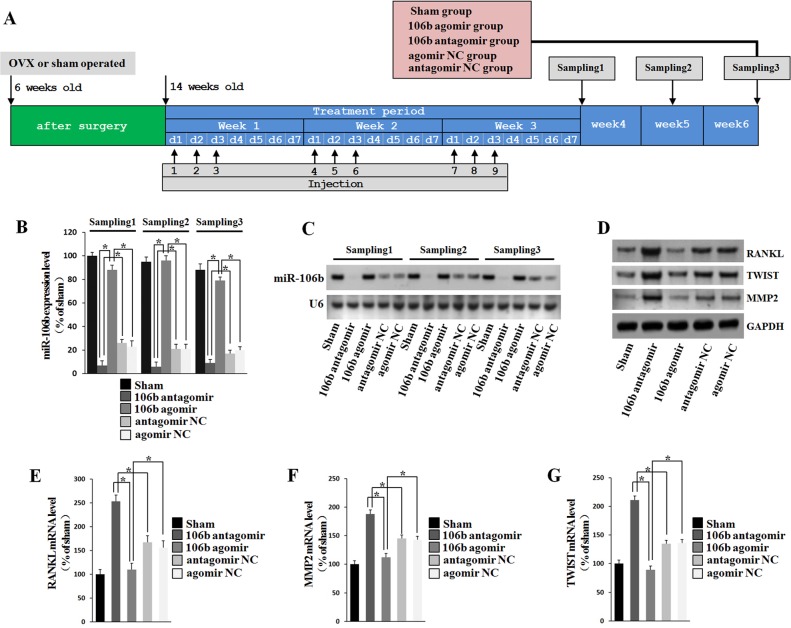
RANKL, MMP2 and TWIST levels are negatively correlated withmiR-106b level in mice **A.** Mice were subjected to OVX operation and rest for two months, and then injected with agomiR-106b, agomirNC, antagomiR-106b or antagomir NC. MiR-106b expression was analyzed by Northern blot at 4, 5 and 6 weeks after the first injection. **B.**, **C.** miR-106b level was measured using qRT-PCR and Northern blot. The result showed that the variation of miR-106b after injection could sustain for at least 6 weeks. **D.** Western blot determination of protein levels of RANKL, MMP2 and TWIST in bone. **E.**-**G.** qRT-PCR determination of mRNA levels of RANKL, MMP2 and TWIST in bone. *P < 0.05.

To further investigate the *in vivo* effect of miR-106b in osteolysis, we selected ovariectomy (OVX) mice to established the animal model, due to the limited proliferation ability and complex composition led to the difficulty of establishing GCT of bone animal models, and the estrogen loss frequently promotes activation of osteoclastic bone resorption [36]. Two months after OVX operation, mice were given agomiR-106b or antagomiR-106b or their controls via a single tail vein injection (Figure 7A). Northern blotting and qRT-PCR showed that after injection, the inhibition or overexpression of miR-106b in bone lasted for at least 3 weeks in coccygeal vertebrae, while the expression of miR-106b in mice that underwent sham operation remained at a relatively high level (Figure 7B, 7C). Six weeks after the first injection, all mice were euthanized and samples were collected for further analysis.

Western Blot was used to measure the protein levels of RANKL, MMP2 and TWIST in the coccygeal vertebrae of the mice. The results showed that the protein levels of ANKL, MMP2 and TWIST were decreased significantly in the antagomiR-106b-treated mice, and increased significantly in the agomiR-106b-treated mice (Figure 7D). The mRNA levels of RANKL, TWIST and MMP2 were also detected by qRT-PCR, and the results showed that the mice treated with agomiR-106b had the minimum levels while mice treated with antagomiR-106b had the maximum levels of these mRNAs among the OVX mice (Figure 7E-7G).

**Figure 8:**
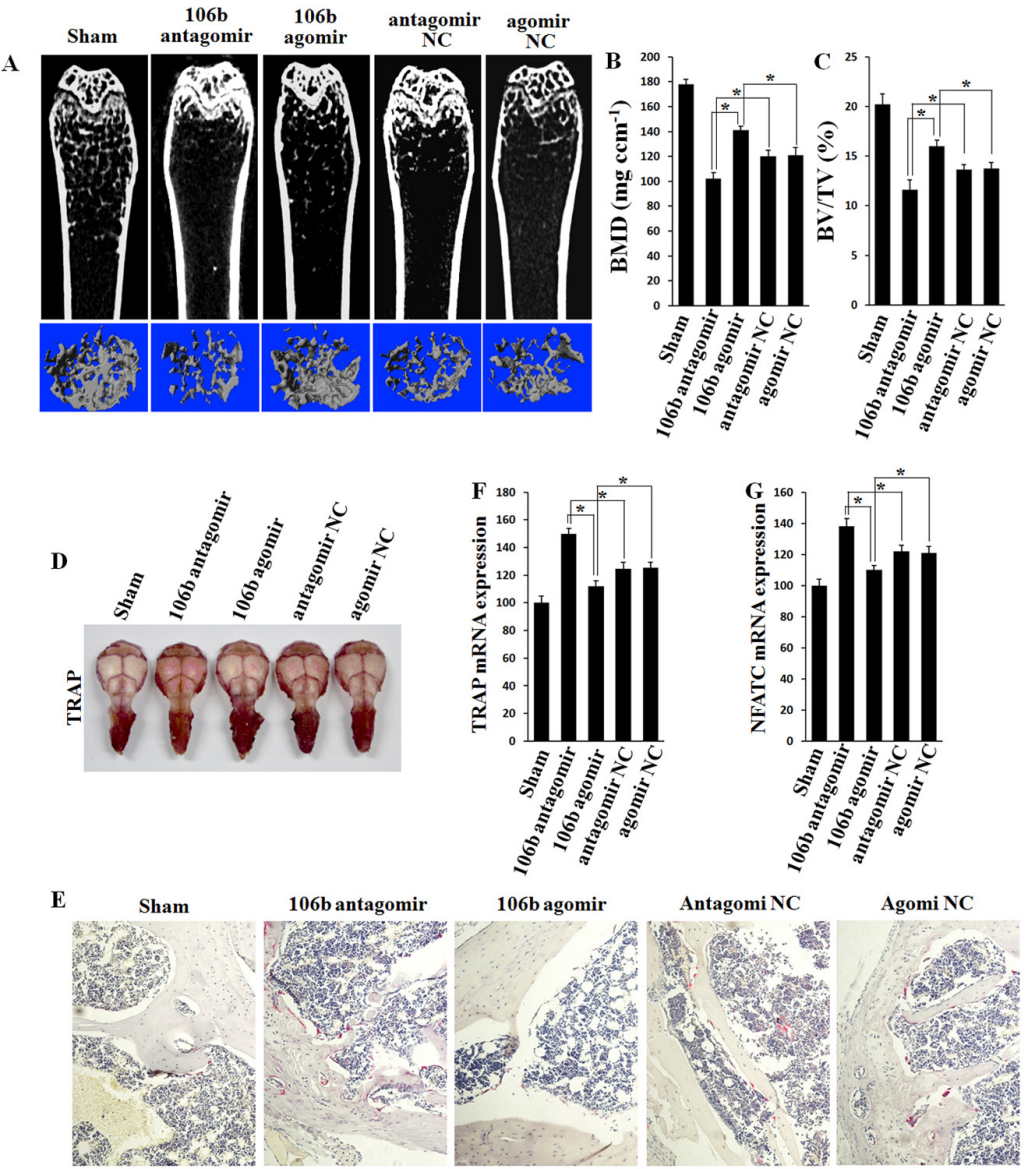
Bone mass is increased and bone resorption is decreased in agomiR-106b treated mice, and the effect of antagomiR-106b is opposite **A.** Micro-CT section and 3D trabecular architecture of distal and middle femoral diaphysis in each group 6 weeks after the first injection. **B.**, **C.** BMD and BV/TV of the femur measured on the results of micro-CT. **D.** TRAP staining of the mouse skull: the deeper the red color, the more the number of osteoclasts. **E.** Light micrographs of TRAP staining performed on trabecular bone section from tibiae. **F.**, **G.** qRT-PCR analysis of TRAP and NFATc1 mRNA levels in bone. *P < 0.05.

MicroCT was performed to detect the quantification of the bone volume/tissue volume ratio (BV/TV), bone mineral density (BMD), trabecular number (Tb.N), trabecular spacing (Tb.Sp) and trabecular thickness (Tb.Th). The results showed that agomiR-106b-treated mice exhibited a significant decrease in femur BMD in comparison with mice treated with con-agomiR-106b, while the BMD of the antagomiR-106b-treated mice presented an obvious increase compared with that of the mice injected mut-antagomiR-106b (Figure 8A, 8B). There was no significant difference in BMD between the two control groups (con-agomir and mut-antagomir). Similarly, overexpression of miR-106b increased BV/TV (Figure 8C) and other bone parameters, including Tb.N, Tb.Sp and Tb.Th (Supplemental Figure 3A-3C), while the absence of miR-106b decreased these parameters.

Histomorphometric analysis was performed to assess the bone resorption parameters, including the number of osteoclasts per bone perimeter (N.Oc/B.Pm) and osteoclast surface per bone surface (Oc.S/BS). The results showed that bone resorption was increased significantly in OVX mice. AgomiR-106b decreased while antagomiR-106b increased N.Oc/B.Pm and Oc.S/BS in the OVX mice (Supplemental Figure 3D, 3E).

We next measured the osteoclast activity by TRAP staining in the mouse skull. The result showed that osteoclasts were significantly activated in antagomiR-106b-injected mice (Figure 8D). By TRAP staining for osteoclasts in bone section from distal femora of mice, we foung that antagomiR-106b increased while agomir-106b decreased osteoclast formation (Figure 8E). In addition, the levels of osteoclast activity markers such as TRAP and NFATc1 mRNA in bone tissue were also elevated in antagomir group, and decreased in agomir group (Figure 8F, 8G). TRAP activity changed similarly in serum (Supplemental Figure 3F). All these results suggest that miR-106b inhibits osteolysis and osteoclastogenesis by targeting RANKL, MMP2 and TWIST *in vivo*.

## DISCUSSION

Giant cell tumor of bone presents with numerous osteoclast-like multinucleated giant cells that are principally responsible for the extensive bone resorption by the tumor [2]. However, GCTSCs were considered as the neoplastic components expressing RANKL and other cytokines that induce giant cell differentiation [9]. Here, we demonstrated that miR-106b was down-regulated in GCT and directly targeted RANKL and suppressed the expression of IL-8, MMP2 and TWIST. By overexpressing miR-106b using TALENs, osteoclastogenesis and osteolysis induced by GCTSCs were significantly suppressed *in vitro*. In addition, by using the GCT model of chick CAM, we found that miR-106b could suppress the expression of RANKL and giant cell formation *in vivo*. We further showed that a single miRNA could negatively dictate osteoclast differentiation and bone resorption by targeting RANKL, MMP2 and TWIST in the OVX mice model. Therefore, we conformed that miR-106b is a suppressor of osteolysis both *in vitro* and *in vivo*.

MiR-106b, located at Chr7, is a member of miR-106b-25 cluster [37]. It is known to regulate stem cell proliferation and neuronal differentiation, causing tumor formation or Alzheimer's disease [37, 38]. Other studies demonstrated that ectopic expression of miR-106b inhibited the expression of P21, RB and Smad3 participated in the regulation of G1-to-S cell cycle transition, thereby leading to excessive proliferation and tumor development [37, 39, 40]. Additionally, miR-106b was reported to induce epithelial-mesenchymal transition (EMT), which is essential for tumor metastasis, by suppressing RUNX3 [41]. Consistent with these mechanisms, miR-106b overexpression has been detected in breast, prostate, gastric, lung and renal cancers [25, 37, 40-42]. However, we found that miR-106b was significantly decreased in GCT. Similarly, Slaby et al [42] demonstrated that miR-106b level was decreased in metastatic renal cell carcinoma and considered a potential predictive marker of early metastasis after nephrectomy. Ni et al [26] also reported a negative correlation between breast cancer bone metastasis and miR-106b levels in orthotopic tumor tissue. Nonetheless, the function of miR-106b decrease in these tumors has not been well defined. Our data indicate that declined miR-106b levels could enhance osteoclast differentiation and bone resorption by targeting RANKL, TWIST and MMP2, which might partly explain the role of miR-106b down-regulation in GCT and bone metastasis.

Previous studies have reported the evidence of direct regulation of IL-8 [27], MMP2 [26] and TWIST [28] by miR-106b in other tumors. In addition, some other studies showed that IL-8, MMP2 and TWIST could promote RANKL production, leading to osteoclast differentiation [26, 43-45]. However, there is still no study reporting the role of regulation of miR-106b in osteoclastogenesis or osteolysis. In the present study, we for the first time demonstrated that miR-106b could suppress IL-8, MMP2 and TWIST expression in GCTSCs and further inhibit bone resorption.

RANKL-induced activation of the NF-kB pathways is essential for osteoclast differentiation [46]. RANKL produced by tumor cells or the bone marrow microenvironment in response to tumor cells played important roles in activation of osteoclastic bone resorption in bone tumors and bone metastases [48]. In bone marrow microenvironment, RANKL was mainly secreted by osteoblast, a cell differentiated from mesenchymal stem cells (MSC), while in GCT, it was mainly produced by GCTSC which also derived from MSC. A few miRNAs has been confirmed to play key roles in osteoblasts [49, 50]. However, the role of miRNAs in GCTSCs has rarely been reported. Our study exhibited the decrease of miR-106b in GCTSCs and indentified RANKL as a new target of miR-106b. In addition, we revealed the binding site of miR-106b in the 3′UTR of RANKL mRNA, and further demonstrated the decrease of RANKL expression induced by over-expression miR-106b in GCTSCs, leading to the inhibition of osteoclastogenesis or osteolysis.

Inhibition of bone resorption has been confirmed to be an effective therapeutic strategy to reduce recurrence of GCT [51]. Bisphosphonate has been widely used in the clinical treatment of GCT for its protective effect against osteolysis [51, 52]. Recently, denosumab, a monoclonal antibody for RANKL, was considered an effective therapeutic option for GCT patients in clinical study [53]. Our results showed an inhibiting effect of miR-106b to osteoclastogenesis or osteolysis *in vitro*. By using the GCT model of chick CAM, we found that miR-106b could suppress RANKL expression and giant cell formation *in vivo*. Due to the limitation of no GCT of bone animal model, we chose OVX model to detect the role of miR-106b during the process of osteolysis. It was found that agomiR-106b could partly recover the osteoporosis induced by OVX via suppressing the expression of RANKL, while antagomiR-106b aggravated osteoporosis. Thus, our study revealed that miR-106b might be a new potential therapeutic target of GCT for its inhibiting effect to osteolysis via targeting RANKL.

In the OVX mice experiments, we also found that miR-106b was significantly down-regulated in this postmenopausal osteoporosis mice model compared with it in mice underwent sham operation, which has never been reported before. RANKL/RANK axis plays a crucial role in osteoporosis and offers important therapeutic targets [54], such as denosumab in the clinical treatment of osteoporosis via targeting RANKL [55]. Our data showed that miR-106b could negatively regulate RANKL in OVX mice and partly inhibit osteoporosis. These results indicate that miR-106b may also play a key role in postmenopausal osteoporosis and be a new possible diagnostic and therapeutic target in this disease, though further investigation is necessary.

In conclusion, our results showed that miR-106b was down-regulated in GCT of bone and negatively regulated the expression of RANKL, IL-8, MMP2 and TWIST, resulting in the inhibition of osteoclastogenesis and osteolysis both *in vitro* and *in vivo*. These results suggest that miR-106b may be a driver of bone resorption via targeting multiple genes including RANKL, and may provide a new possible approach to the diagnosis and treatment of GCT and other diseases associated with bone destruction, such as postmenopausal osteoporosis and bone metastasis.

## MATERIALS AND METHODS

### Clinical samples

The study population comprised 30 Chinese GCT patients aged 12-56 years who underwent resection for primary GCT in our hospital between July 2011 and July 2012. Patients with metastases and those with hepatopathy, nephropathy, diabetes mellitus, hematologic diseases, rheumatoid arthritis, and diseases of the thyroid or parathyroid were excluded from the analysis, knowing that these conditions may affect bone metabolism. Patients who received radiotherapy/chemotherapy, or used bone-modifyingagents such as zoledronic acidbefore surgery were also excluded. Primary GCT tissues were isolated from tumor samples derived from tumor resections. The non-tumor infected cancellous bones from the same GCT patients were used as normal controls. The tissues were snap-frozen and stored in liquid nitrogen within two hours after surgical excision. The clinical study was approved by the Ethnic Committee of Changzheng Hospital of the Second Military Medical University (Shanghai, China), and written informed consent was obtained from all participants.

### miRNA microarray assay

Total RNA was extracted from the GCT tissue (*n* = 17) and cancellous bone (*n* = 4) using Trizol (Invitrogen, Carlsbad, CA, USA). Microarrays were performed by utilizing the miRCURY LNA™ microRNA Array (Version 14.0 Exiqon, Vedbaek, Denmark). After RNA measurement on the NanoDrop2000 (Thermo Scientific, Wilmington, USA) instrument, the samples were labeled using the miRCURY™ Array Power Labeling kit (Cat #208032-A, Exiqon) and hybridized on the miRCURY LNA™ Array (v.14.0). After the labeling procedure, the RNA sample was mixed pair wise and hybridized to the mercury LNA™ Array version 14.0 (Exiqon). The hybridization and subsequent wash steps were performed according to the miRCURY LNA™ array manual. The microarray slides were scanned using the Axon Gene Pix 4000B scanner (Axon Instruments, USA) and image analysis was carried out using the Gene Pix pro V6.0 software (Axon Instruments, USA). Statistical comparisons were performed by analysis of variance (ANOVA).

### Cell lines and culture

For primary cell culture, bone marrow-derived macrophages (BMM) isolated from C57/BL6 mice and GCTSCs isolated from GCT samples were cultured as described previously [4]. BMSCs were isolated from bone marrow of the GCT patients as described previously [56]. This had been approved by the Ethnic Committee of Changzheng Hospital of the Second Military Medical University and specimens were taken with patients' written consent. HEK293 (ATCC, Manassas VA) and MG63 (ATCC, Manassas VA) cells were maintained in DMEM (GIBCO) supplemented with 10% fetal bovine serum (HyClone) in the cell incubator (37 °C, 5% CO2). BMM and GCTS cells were maintained in α-MEM (GIBCO) supplemented with 10% fetal bovine serum.

### qRT-PCR for mRNA and miRNA analysis

qRT-PCR for mRNA was performed by iTaq™ Universal SYBRGreen (Bio-Rad Laboratories, CA, USA) on 7900HT Fast Real-Time PCR System (Life Technologies Corporation, USA). All PCR primers are listed in Supplemental Table 2. The expression of miRNA-106b and U6 was examined by TaqManmiRNA Assay system (Life Technologies Corporation, USA).

### *In situ* hybridization (ISH) and immunofluorescence (IF)

ISH was performed using the miR-106b locked nucleic acid probe (5′-digoxigenin-ATCTGCACTGTCAGCACTTTA-3′-digoxigenin) and the microRNA ISH Optimi-zation Kit (Exiqon, Vedbaek, Denmark) according to the manufacturer's instructions, as described previously [23]. The signals of IF were examined with a BX51 fluorescence microscope (Olympus).

### Western blot and ELISA

For western blot, the antibodies against RANKL (ab124797) were purchased from Abcam. The antibodies against IL-8 (sc-73321), MMP2 (sc-53630) and TWIST (sc-15393) were purchased from Santa (Santa Cruz, USA). ELISA assay was performed with Uscn (Uscn Life Science Inc., Wuhan, China) ELISA kit for human RANKL (SCA855Hu) following the manufacturer's instructions.

### Histology and immunohistochemistry

The tissue sections were stained with hematoxylin-eosin and by immunohistochemistry using an indirect immunoperoxidase technique, with the antibodies against RANKL, IL-8, MMP2, TWIST, RANK (sc-34249, Santa Cruz, USA) and OPG (sc-8468, Santa Cruz, USA). Results were analyzed by standard light microscopy.

### Plasmids

RANKL 3′-UTR constructs were PCR amplified using cDNA encoding RANKL (NM_033012.3) as templates, and subcloned into pGL3-Basic vector using In-Fusion™ advantage PCR cloning Kit (Clontech, USA) for Luciferase reporter gene assay. The construction of the RANKL 3′-UTR mutant was done by PCR using the Quickchange™ site directed mutagenesis kit from Qiagen (Qiagen, USA).

### Transfection of agomiR-106b and antagomiR-106b

The agomiR-106b, antagomiR-106b and negative controls of miR-106b were purchased from RiboBio (RiboBio, Guangzhou, China). Cells were transfected with agomiR-106b, antagomiR-106b and negative controls at a final concentration of 50 nM. The FuGENE HD transfection agent (Promega, USA) was used according to the manufacturer's instructions.

### Luciferase reporter assay

Dual luciferase assays were conducted in a 24 well plate format. pGL-RANKL 3′ UTR report / pGL-RANKL 3′ UTR Mutant report + TK100 Renilla report were transfected into 70% confluent HEK293 cells, along with agomiR-106b, antagomiR-106b (antago-miR-106b) or each control. After 48-h transfection, firefly and renilla luciferase were quantified sequentially using the Dual Luciferase Assay kit (Promega, USA) following the manufacturer's recommendations.

### Construction of stable cell lines using TALENs

The plasmid of TALENs targeting the PPP1R12C (the AAVS1 locus) was constructed using Fast TALETM TALEN Assembly kit from SiDanSai biotechnology (SiDanSai, China). The PPP1R12C locus homologous sequences were PCR amplified using genomic DNA extracted from HEK293 cells as template, and cloned in pEASY-T1 vector(TransGen, China) as Donor-1. The following primer sets were used: forward, 5′-ATGCCGTCTTCACTCGCTGGGTT-3′; reverse,5′-CTCCTGGGCTTGCCAAGGACTCAA-3′. Then, Donor-HindIII mutant vector was constructed using Donor-1 vector as template by PCR, and using Quickchangesite directed mutagenesis kit using following primer sets: forward, 5′-GGCCACTAGGGACAAGCTTGGTGACAGAA-3′; reverse, 5′-GCTTGTCCCTAGTGGCCCCACTGTGG-3′. After that, human cytomegalovirus (CMV) immediate early promoter gene, enhanced green fluorescent protein (EGFP) gene, pri-mir106 gene, SV40 early mRNA polyadenylation signal gene were inserted into Hind III site from Donor-Hind III vector in turn. All clones were constructed using In-Fusion TM advantage PCR cloning Kit (Clontech, USA).

The two TALENs and corresponding Donor plasmids were transfected into GCTSCs. After selection with puromycin, resistant colonies with green fluorescence were picked up, and examined by genomic PCR (the following primers were used: forward, 5′-ATGCCGTCTTCACTCGCTGGGTT-3′; reverse, 5′-CTCCTGGGCTTGCCAAGGACTCAA-3′.) and digestion with EcoRI restriction enzyme.

### Sourthern blot

Each sample of total DNA was extracted from OE-106b and OE-Ctrl cells using the Puregene kit (Qiagen, Valencia, CA). Approximately 10 μg of total DNA were digested with restriction endonucleases EcoRV and EcoRI for 8 h at 37 °C. DNAs were denatured and separated by electrophoresis on a 0.8% agarose gel and transferred onto BioBond Plus nylon membrane (Sigma, USA). Blots were hybridized with a α32P dCTP-(3000 Ci/mmol) (PerkinElmer) labeled probe corresponding to the 411 bp left arm of PPP1R12C locus DNA generated with the Random Primed DNA Labeling Kit (Roche, Mannheim, Germany). Hybridizations were performed overnight at 50 °C.

### TRAP staining assays, actin ring-formation and osteoclastogenesis assays

For TRAP staining, cells were fixed and stained using the TRAP activity kit (Sigma, USA). TRAP-positive multinucleated cells containing three or more nuclei were counted as mature osteoclasts. For actin ring formation assay, cultured BMM cells were first fixed with 4% PFA in PBS for 10 min, permeabilized with 0.1% Triton-X 100 in PBS for 5 min, and then incubated with rhodamine-conjugated phalloidin (Molecular Probes, Eugene, OR, USA). The ring structure of F-actin dots that indicates osteoclastogenesis was observed under a fluorescent microscope [34]. For osteoclastogenesis assay, BMMs were seeded on a dentin slice and cultured with the conditional mediumcontaining MCFS (10 ng/mL) for 7 days, with the medium changed every 2 days.

### The chick chorio-allantoic membrane (CAM) assay

A short-term model of GCT *in vivo* was set up in the chick CAM as described previously [35]. Fertilized white leghorn chicken eggs (Valo-SPF eggs, LohmannTierzucht GmbH, Cuxhaven, Germany) were incubated at a humidity of 70% and 37°C. At embryonic day 3, 2-3 ml albumen was removed with a syringe. After 10-day incubation, small plastic rings made of Thermanox™ cover discs were placed on the CAM. After gentle laceration of the CAM surface, 20 μl re-suspended tumor suspension with 2nmol agomiR-106b or PBS was deposited into the rings (8 eggs per group). After 24h suspension grafting, a solid tumor became apparent. Then 2nmol agomiR-106b or PBS were injected into the tumor every two days. Until day 16, CAMs were measured and collected for further analysis. All embryos that died before day 16 were excluded from further analysis.

### Mice

Thirty-six female WT C57BL/6 mice aged 6 weeks were equally randomized to 6 groups, of which 5 groups underwent OVX operation, and the remaining group underwent sham operation. Two months after operation, the 5 OVX groups of mice respectively received 10nmol/per mouse of agomiR-106b, con-agomiR-106b, mut-antagomiR-106b, antagomiR-106b or 0.2ml PBS through the tail vein on day 1-3 for 3 consecutive weeks. A section of coccygeal vertebrae were get out from all the mice before injection and at 3, 4.5 and 6 weeks after the first injection (Figure 7A). Six weeks after the first injection, mice were euthanized. Bone and serum samples were collected for further experiments. The agomir, con-agomir, mut-antagomir and antagomir were all purchased from RiboBio (RiboBio, Guangzhou, China). All procedures involving the mice were approved by the Animal Management Committeeof the Second Military Medical University.

### Northern blot

20μg RNA was loaded onto 15% ureapolyacrylamide gel with 0.5×Tris borate-EDTA for electrophoresis. Then separated RNA was transferred to a Hybond-N+ nylon membrane (Amersham Biosciences) using a semidry transfer cell (Bio-Rad). Hybridization was performed according to a standard protocol. P-Labeled oligonucleotide probes complementary to the mature miR-106b and U6 were used in hybridization. The blots were processed using Typhoon™ FLA 7000 biomolecular imager.

### Micro-CT analysis

Bone samples were removed and the distal left femur of each mouse was scanned by microCT (SCANCO viva 4.0). The Image-Pro+ 7.0 and SCANCO microCT software packages were used to analyze BMD and several structural parameters of the proximal femur, including BV/TV, Tb.Th, Tb.N, and Tb.Sp.

### Statistics

SPSS 19.0 statistical software (SPSS Inc., Chicago, IL) was used for statistical analysis. All data are presented as mean ± standard error of the mean (SEM). Statistics were assessed using both Student t test and ANOVA, assuming double-sided independent variance. All experiments were repeated at least three times, and representative experiments are shown. *P* values of < 0.05 were considered statistically significant.

## SUPPLEMENTARY MATERIAL FIGURES AND TABLES


