# Supplementary material for: MicroRNA-106b inhibits osteoclastogenesis and osteolysis by targeting RANKL in giant cell tumor of bone
Source: Oncotarget. 2015 May 22;6(22):18980–96. doi: 10.18632/oncotarget.4223 (PMC4662469; doi:10.18632/oncotarget.4223)
Supplement: Supplementary file 1 [file oncotarget-06-18980-s001.pdf]

MicroRNA-106b inhibits osteoclastogenesis and osteolysis by targeting RANKL in giant cell tumor of bone

Supplementary Material

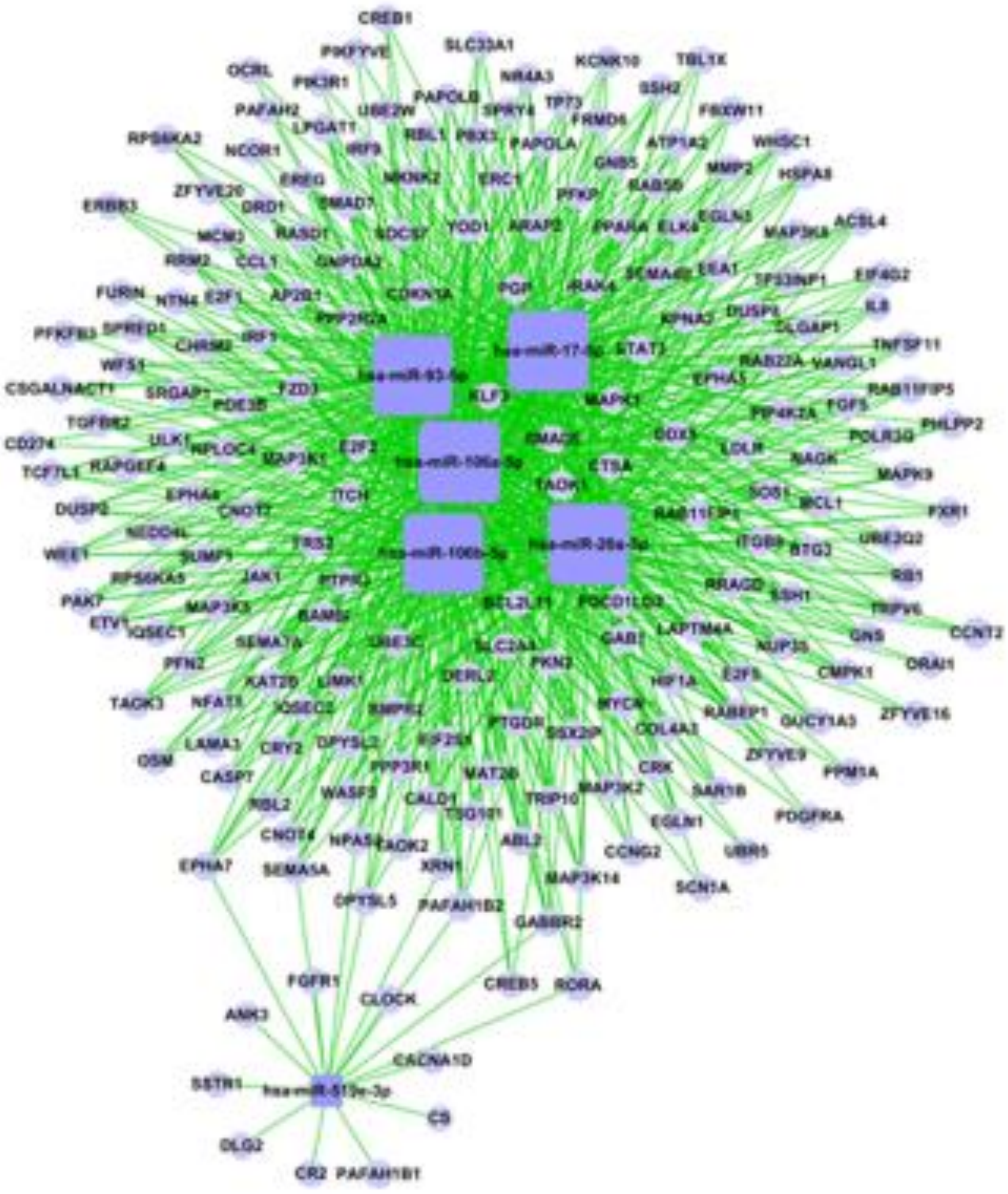

Supplemental Figure 1: The result of bioinformatic analysis indicates that miR-106b may play a key role in GCT.

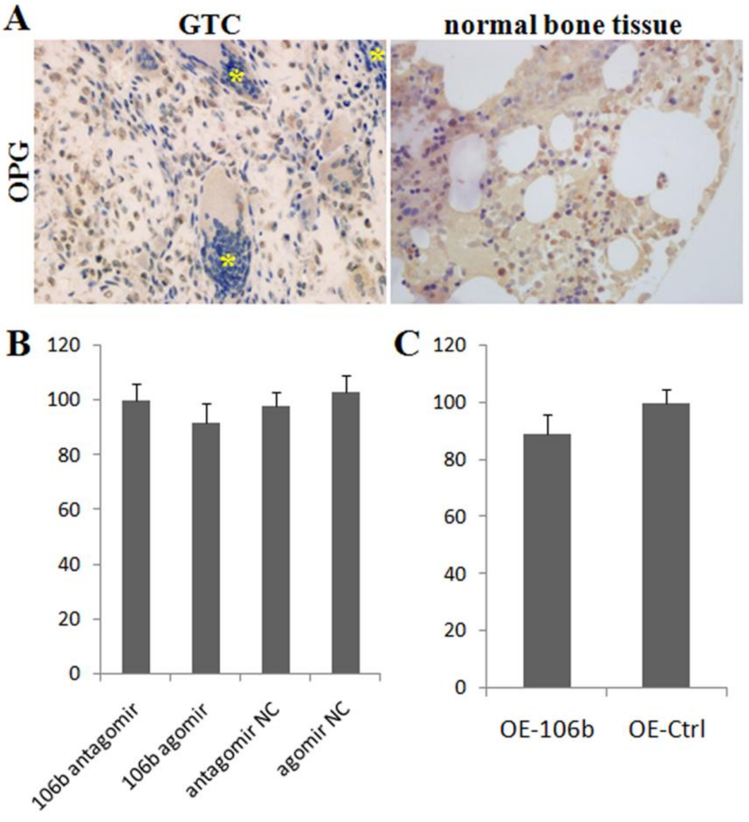

Supplemental Figure 2: MiR-106b showed no clear effect on OPG expression. (**A**) IHC staining of OPG in human GCT tissue and normal bone tissue specimens. (**B**) qRT-PCR measurement of mRNA levels of OPG after the transfection of agomiR-106b or antagomiR-106b. (**C**) qRT-PCR measurement of mRNA levels of OPG in OE-106b and OE-ctontrol cells

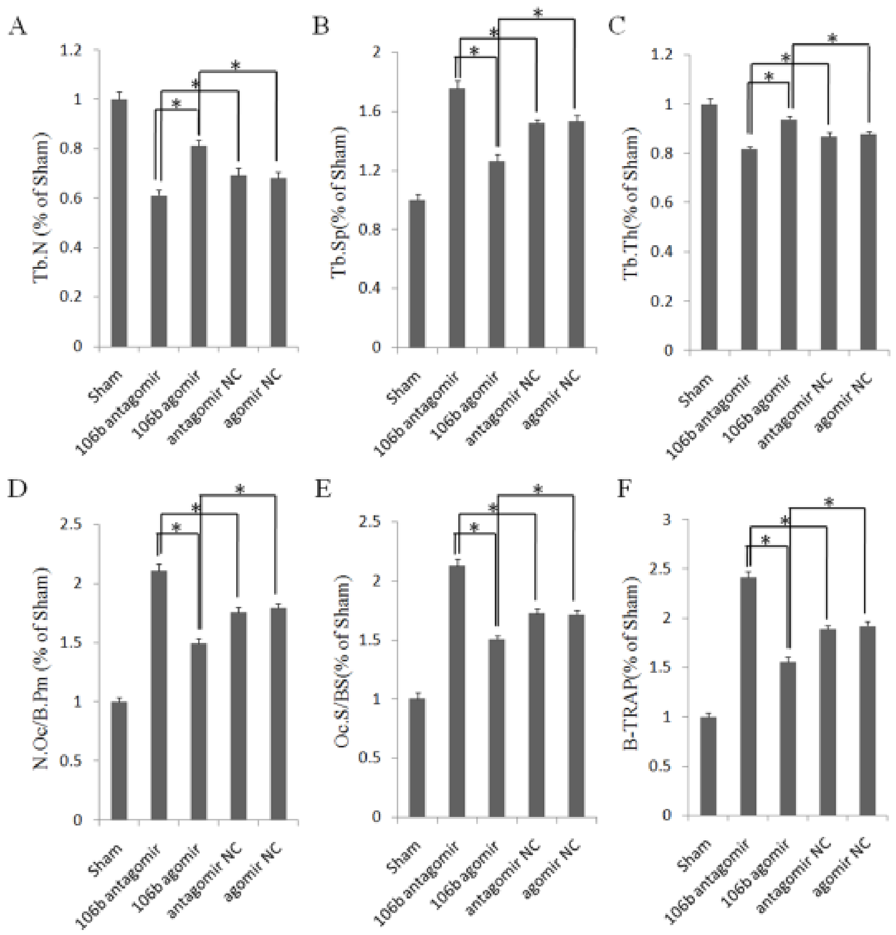

Supplemental Figure 3: Bone mass is decreased and osteoclast activity is increased in antagomiR-106b treated mice, and the effect of agomiR-106b is opposite. (**A**) Tb.N, (**B**) Tb.Sp and (**C**) Tb.Th of femurs were measured by microCT. (**D**) N.Oc/B.Pm and (**E**) Oc.S/BS were measured by histomorphometric analysis. (**F**) Serum concentrations of TRAP were detected. Data are shown as mean ± SD. *P<0.05.

Supplemental Table 1: Clinical features of the GCT patients in this experiment

Patients	Sex	Age (years)	Disease history (months)	Tumor size (cm)	Tumor site	Primary/ recurrence	Surgical operation
1	Female	16	1	7	Sacrum	Primary	Total resection
2*#	Female	28	6	9	Sacrum	Primary	Total resection
3	Female	41	6	6	Sacrum	Primary	Total resection
4*	Female	37	72	7	Thoracic	Recurrence	Total resection
5	Female	37	8	4	Lumber	Primary	Total resection
6*	Female	31	72	5	Thoracic	Recurrence	Subtotal resection
7*	Male	36	8	6	Sacrum	Primary	Total resection
8	Female	26	10	3	Thoracic	Primary	Total resection
9*	Female	23	8	10	Sacrum	Recurrence	Subtotal resection
10*	Male	30	1	5	Thoracic	Recurrence	Total resection
11	Male	31	7	9	Thoracic	Primary	Total resection
12*#	Male	24	5	2	Cervical	Primary	Total resection
13*	Female	21	12	7	Cervical	Recurrence	Subtotal resection
14	Male	35	6	7	Sacrum	Primary	Total resection
15*	Female	13	6	5	Cervical	Primary	Total resection
16	Male	29	6	6	Cervical	Primary	Total resection
17*	Male	49	144	8	Cervical	Recurrence	Total resection
18	Female	30	6	7	Thoracic	Primary	Total resection
19*	Male	51	10	9	Lumber	Recurrence	Total resection
20	Female	12	12	5	Right femur	Primary	Total resection
21	Female	48	48	8	Lumber	Recurrence	Total resection
22*#	Female	44	60	7	Lumber	Recurrence	Total resection
23*	Male	56	24	15	Sacrum	Primary	Total resection
24	Female	36	2	6	Thoracic	Recurrence	Total resection
25*	Male	37	108	10	Thoracic	Primary	Subtotal resection
26	Female	32	6	12	Sacrum	Primary	Subtotal resection
27*	Female	29	60	9	Thoracic	Recurrence	Total resection
28*	Male	40	9	6	Thoracic	Recurrence	Total resection
29*#	Female	47	24	8	Thoracic	Primary	Total resection
30	Male	28	16	13	Sacrum	Recurrence	Subtotal resection

* indicats cases involved in MiRNA microarray assay of GCT tissues; # indicats cases participated in MiRNA microarray assay of cancellous bone as normal controls.

Supplemental Table 2: Primers of qRT-PCR assays for mRNAs

Gene	Acc. No	Primer sequence(5' to 3')	Size
RANKL （human）	NM_003701.3	F: GCTTGAAGCTCAGCCTTTTGCTCAT	25
		R: GGGGTTGGAGACCTCGATGCTGATT	25
RANKL （mouse）	NM_011613.3	F: CATCAATGCTGCCAGCATCC	20
		R: CTGAAGATAGTCTGTAGGTACGC	23
TWIST(human)	NM_000474.3	F: CGACGACAGCCTGAGCAACA	20
		R: CCACAGCCCGCAGACTTCTT	20
TWIST(mouse)	NM_011658.2	F: ACGCAGTCGCTGAACGAGGC	20
		R: GTACAGGAAGTCGATGTACC	20
IL-8(human)	NM_000584.3	F: CTTGGCAGCCTTCCTGATTTCT	22
		R: GTTTTCCTTGGGGTCCAGACAG	22
MMP2(human)	NM_004530.4	F: GTTCATTTGGCGGACTGT	18
		R: AGGGTGCTGGCTGAGTAG	18
MMP2(mouse)	NM_008610.2	F: CAAGTTCCCCGGCGATGTC	19
		R: TTCTGGTCAAGGTCACCTGTC	21
TRAP(mouse)	NM_001102405.1	F: GAACCGTGCAGACGATGG	18
		R: GGAAGTTCCAGCGCTTGG	18
NFATc1(mouse)	NM_016791.4	F: AGTCATCGGCGGGAAGAAG	19
		R: CCATTGGCAGGAAGGTACG	19
GAPDH(human)	NM_002046.5	F: GGAGTCCACTGGCGTCTTCA	20
		R: GGGGTGCTAAGCAGTTGGTG	20
GAPDH(mouse)	NM_001289726.1	F: GGTGAAGGTCGGTGTGAACG	20
		R: CTCGCTCCTGGAAGATGGTG	20
